# Association between Serum Gamma-glutamyl transferase and Intracranial Arterial Calcification in Acute Ischemic Stroke Subjects

**DOI:** 10.1038/s41598-019-56569-7

**Published:** 2019-12-27

**Authors:** Tao Yao, Jing Li, Qi Long, Gang Li, Yanbin Ding, Qin Cui, Zhichao Liu

**Affiliations:** 1 Department of Neurology, Renmin Hospital of Wuhan University, Wuhan, China; 2 Emergency Department, Hubei Provincial Hospital of Traditional Chinese Medicine, Wuhan, 430061 China; 3 Hubei Province Academy of Traditional Chinese Medicine, Wuhan, China; 4 Department of Neurology, Hubei Provincial Hospital of Traditional Chinese Medicine, Wuhan, 430061 China

**Keywords:** Neuro-vascular interactions, Stroke

## Abstract

Intracranial artery calcification (IAC) is an important risk factor for cerebral infarction and a key biomarker for intracranial artery stenosis. Gamma-glutamyl transferase (GGT) has been independently associated with increased cardiovascular events and coronary calcification. Our study assessed whether GGT is an independent factor for IAC in acute ischemic stroke (AIS) patients. This cross-sectional study involved a total of 754 patients with AIS (mean age: 65 ± 13.2 years). All the patients had received brain computed tomography angiography (CTA) examination to evaluate IAC. Further, serum GGT levels and other biochemical parameters were analyzed. The average GGT level in patients who died was also significantly increased (37.0 ± 26.8 vs 29.0 ± 21.5 U/L, p = 0.012). Partial correlation analysis showed that serum GGT levels were associated with NIHSS score at admission after adjustment for age and gender was considered (r = 0.150, p = 0.001). Logistic regression analysis showed that serum GGT levels independently predicted all-cause mortality (OR = 1.036, 95% CI: 1.014–1.060, p = 0.002), NIHSS scores (β = 0.051, 95% CI: 0.020–0.082, p = 0.001) and IAC scores (β = 0.006, 95% CI: 0.003–0.014, p = 0.005) in male patients. Each SD (standard deviation) increase of serum GGT levels was also associated with risk of all-cause mortality (OR = 2.272, 95% CI: 1.364–3.787, *P* = 0.002). GGT levels in patients with severe IAC were significantly elevated (37.6 ± 33.6 vs 28.6 ± 19.2, p < 0.001). However, serum GGT levels could not independently predict the severity of IAC in AIS patients. Our study identified that serum GGT levels were significantly elevated in patients who died, and the GGT levels had a certain association with the risk of death and IAC in male patients.

## Introduction

Intracranial artery calcification (IAC) can be easily identified through a computed tomography (CT) head scan, thus enabling it as a potential non-invasive biomarker. A large number of accumulated evidences in patients, especially in Asian population, suggest a correlation between IAC and ischemic stroke, decreased cognitive ability, and other vascular diseases^1–3^. IAC is an important risk factor for cerebral infarction and a key biomarker for intracranial artery stenosis.

The degree of IAC is related to the condition of atheromatous plaques. The coronary calcification score is currently widely used to assess the risk of cardiovascular events in patients. Previously, we found that serum gamma-glutamyl transferase (GGT) levels were independently associated with progression of coronary calcification in patients with type 2 diabetes mellitus (T2DM)^4^. GGT is a key enzyme that hydrolyzes the antioxidant glutathione. This hydrolysis reaction produces many pro-oxidants including reactive oxygen species (ROS) and oxidized low-density lipoprotein (OX-LDL), which can increase the formation and development of atherosclerotic plaques^5^. Previous studies found that blood GGT activity were independent biomarkers of coronary calcification^6,7^. However, the relationship between GGT and IAC has not been further evaluated.

A large number of studies have shown that GGT could be used as a risk factor for cardiovascular diseases (CVD) and could allow to independently predict all-cause mortality and fatal cardiovascular events in patients with coronary heart diseases (CHD)^8,9^. Jousilahti *et al*., reported that the GGT enzyme is associated with ischemic stroke^10^. A study with a small sample size showed that elevated GGT levels were associated with infarct size in acute ischemic stroke (AIS)^11^. Furthermore, Tu W. J. *et al*., demonstrated that GGT was independently associated with all-cause and CVD mortality in patients with ischemic stroke^12^. In this study, the main objectives are to assess the relationship between GGT and IAC and to assess whether GGT levels can independently predict short-term mortality in patients with AIS.

## Methods

### Subjects

The present study was performed in Department of Neurology, Renmin Hospital, Wuhan University, China. All patients referred for AIS between January 2017 and December 2018 were screened. Each patient received brain CT and/or MR imaging and the diagnosis of ischemic stroke was based on the agreement of at least two neurologists. The present study was in line with the Declaration of Helsinki. This study was approved by the Human Research Ethics Committee of Renmin Hospital of Wuhan University (Wuhan, China, No. 2016H11018). Each participant received written informed consent. We have signed the consent of each study participant. All results were anonymous. No separate results are displayed, only group statistics are provided.

According to AHA/ASA stroke guidelines, 913 adult patients above 35 years of age with initially AIS were admitted to the University Affiliated Hospital within three days of onset of symptoms. Patients with a history of stroke or old cerebral infarction on CT/MRI images were excluded from the initial screening. In this study, we used the WHO definition to determine whether patients were smoking or drinking. Smoking is defined as smoking more than one cigarette a day for six months. Drinking is defined as drinking at least once in the past 30 days. These information were obtained directly from study patients or their hospital medical records.159 patients were excluded from the study: 30 patients did not undergo computed tomography angiography (CTA) examination, 63 patients were missing follow-up results, 31 patients had a history of chronic liver disease and significant abnormal liver function, and 35 patients did not receive National Institutes of Health Stroke Scale (NIHSS) score at admission. Therefore, a total of 754 patients were finally included in this cross-sectional study.

### Biochemical measurements

All participants included in the study had accepted head CT, electrocardiogram (ECG), magnetic resonance imaging (MRI), transthoracic echocardiography (TEE) and standard laboratory examinations at admission. Blood samples were collected from the antecubital vein in a vacuum tube containing EDTA. Patients were asked to fast for 8 hours, and levels of liver enzyme, blood lipid profiles, renal biochemical index, fasting plasma glucose (FPG), homocysteine (Hcys) and glycosylated hemoglobin (HbA1c) levels were measured by standard methods. Serum GGT activity was analyzed with an enzyme kinetic assay (Olympus AU600; Roche Diagnostics, Shanghai, China), based on the recommendation of European Committee for Clinical Laboratory Standards^13^. Serum GGT activity was measured at 37 °C and recalculated to 25 °C and were expressed as units per liter (U/L). Between-analyzer differences were <5%. The reference range for GGT activity was 0 to 50 U/L^13^. The following variables were collected in the AIS phase: age, gender, causes of ischemic stroke (according to TOAST criteria)^14^, previously confirmed or found risk factors such as hypertension, diabetes, BMI, coronary heart disease (CHD), peripheral artery disease (PAD), smoking, and long-term alcohol consumption.

### Assessment of IAC score

All patients underwent multidetector brain CTA using a 64-slice spiral CT device (GE Healthcare, Milwaukee, WI, USA). The parameters were as follows:120 kVp, 140 mA, 0.9-mm section thickness, 0.9-mm slice acquisition interval, and intravenous administration of 80 mL of iohexol at a rate of 5.0 mL/s^15^. Bone window CT images covered the whole brain (from the skull base to the vertex), to identify the presence of IAC. IAC lesions were defined as high density lesions with a median density greater than 130 Hounsfield units^16^. We used the 5-point scale semi-quantitative scoring system proposed by Babiarz *et al*. to evaluate IAC^17^. We selected the highest calcification score for each cerebral artery and added the calcification score of all evaluated artery to get the patient’s total calcification score. We distinguished the severity of IAC according to the total calcification score. 0 score for absent, 1–4 for mild, 5–8 for moderate, and 9–12 for severe degree^18^. Two experienced neurologists independently reviewed the images from CT angiography and graded the degree of IAC.

### Statistical analyses

We used SPSS version 22.0 (SPSS Inc, Chicago, Illinois) statistical software for statistical analysis. Comparison of continuous variables in normal distribution by Student’s t-test and ANOVA. A log_2_ transformation of non-normal distributed variables was used to reduce the influence of skew. The categorical variables were analyzed by chi-squared test. Patients were divided into four quartile groups according to the serum GGT levels. Adjusted for age and gender, Partial Spearman correlation coefficients were used to clarify the association between serum GGT with Hcys and NIHSS score. We used logistic regression analysis to examine the relationships between serum GGT levels with all-cause hospital mortality risk, NIHSS score and IAC score. These unified variables of age, hypertension, diabetes mellitus, smoking, drinking, CHD, DBP, TC, TG, Hcys, HbA1c and uric acid (UA) were adjusted in logistic regression analyses. Further analyses were performed to determine multivariable-adjusted odds ratios (ORs) of all-cause hospital mortality risk and severe IAC for the quartiles of serum GGT levels in male AIS patients. In additional, log transformed of GGT was analyzed for *P*_trend_. Z scores for GGT standardization was analyzed for Per 1 SD (standard deviation) increase of ORs. *P* < 0.05 was considered to have significant difference.

## Results

**Figure 1:**
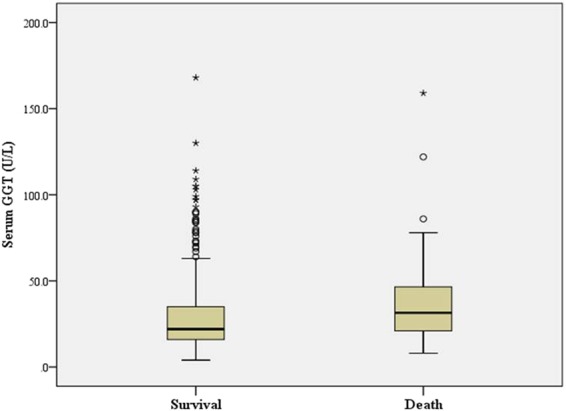
Box and whisker plots illustrate the mean and interquartile ranges of serum GGT levels in patients with survival and death. Open circles and asterisks show outliers.

A total of 754 AIS patients with an average age of 65 ± 13.2 years, including 515 males were recruited in the study. We reviewed the 60-day follow-up of patients after admission and found that 85 patients died during hospitalization and follow-up. The median GGT level included in the study was 23.0 (16.0–36.0) U/L. The mean IAC score was 3.77 ± 3.50. The mean admission NIHSS score was 6.55 ± 6.34. According to TOAST classification, 284 patients were grouped into type 1 infarction, 313 patients were type 2, 63 patients were type 3, 76 patients were type 4 and 18 patients were type 5. Analysis of variance showed that the NIHSS score and mortality rate of the patients in the highest GGT quantile group were significantly higher than those of the other three groups (Table 1). The average GGT level in patients who died was also significantly higher than that of the survived patients (37.0 ± 26.8 vs 29.0 ± 21.5 U/L, p = 0.012, Fig. 1). However, there were no significant differences in IAC scores between patients in different quantile groups (Table 1).

**Table 1:** Clinical and laboratory data for 754 patients divided into quartiles of serum GGT levels.

	Quartile 1 ≤16 U/L	Quartile 2 17–23 U/L	Quartile 3 24–36 U/L	Quartile 4 ≥37 U/L	*P* value
Age (years)	69.7 ± 13.4	66.4 ± 11.5	63.7 ± 12.7	61.1 ± 13.7	<0.001
Male, n (%)	94 (49.6%)	134 (71.4%)	145 (76.8%)	142 (75.4%)	<0.001
BMI (kg/m^2^)	22.85 ± 2.59	22.81 ± 2.67	23.78 ± 2.71	23.92 ± 2.94	<0.001
Smoking, n (%)	42 (22.3%)	73 (38.9%)	80 (42.7%)	77 (40.7%)	0.004
Drinking, n (%)	23 (12.4%)	48 (25.4%)	51 (27.0%)	48 (25.4%)	0.023
HT, n (%)	126 (66.9%)	135 (71.4%)	131 (69.6%)	129 (68.6%)	0.894
DM, n (%)	50 (26.7%)	49 (26.2%)	66 (34.8%)	67 (35.6%)	0.226
CHD, n (%)	23 (12.4%)	15 (7.9%)	20 (10.7%)	22 (11.9%)	0.606
SBP, mmHg	149.0 ± 25.1	153.2 ± 26.9	147.6 ± 25.5	150.9 ± 26.8	0.370
DBP, mmHg	82.4 ± 13.9	83.6 ± 14.7	84.8 ± 15.7	89.2 ± 16.3	0.004
HR (bpm)	76.8 ± 13.2	74.8 ± 11.5	79.0 ± 13.4	80.2 ± 15.2	0.01
ALT (U/L)	14.9 ± 8.7	17.0 ± 7.1	21.0 ± 9.5	26.2 ± 16.2	<0.001
AST (U/L)	21.2 ± 8.8	19.6 ± 5.6	22.2 ± 9.0	27.7 ± 14.8	<0.001
Cr (umol/L)	75.3 ± 28.6	77.6 ± 34.8	75.3 ± 25.9	77.5 ± 26.2	0.883
UA (umol/L)	343.6 ± 101.0	371.7 ± 124.9	379.1 ± 102.2	385.5 ± 135.2	0.032
FBG (mmol/L)	6.6 ± 3.1	6.8 ± 3.5	7.5 ± 3.7	7.9 ± 3.8	0.019
TC (mmol/L)	4.20 ± 0.93	4.50 ± 1.00	4.59 ± 1.13	4.68 ± 1.07	0.004
TG (mmol/L)	1.39 ± 0.75	1.49 ± 0.75	1.87 ± 1.58	2.05 ± 1.75	<0.001
HDL-C (mmol/L)	1.12 ± 0.38	1.13 ± 0.45	1.08 ± 0.33	1.18 ± 0.99	0.619
LDL-C (mmol/L)	2.36 ± 0.76	2.65 ± 0.81	2.62 ± 0.96	2.65 ± 0.87	0.028
sdLDL-C (mmol/L)	0.73 ± 0.31	0.89 ± 0.41	1.05 ± 0.56	0.98 ± 0.43	<0.001
ApoA1 (g/L)	1.29 ± 0.21	1.30 ± 0.20	1.33 ± 0.22	1.33 ± 0.26	0.424
ApoB (g/L)	0.86 ± 0.29	0.90 ± 0.23	0.94 ± 0.22	0.95 ± 0.23	0.063
HbA1c (%)	6.44 ± 1.51	6.90 ± 1.90	7.13 ± 1.86	7.14 ± 1.91	0.033
D-dimer (mg/L)	1.35 ± 2.06	0.95 ± 1.90	0.99 ± 1.92	1.70 ± 3.18	0.06
Hcys (umol/L)	19.0 ± 12.6	18.6 ± 11.7	18.0 ± 8.7	20.8 ± 15.5	0.423
IAC score	3.41 ± 3.46	3.67 ± 3.35	3.79 ± 3.51	4.21 ± 3.68	0.358
Death, n (%)	15 (8.3%)	12 (7.1%)	21 (11.6%)	37 (20.3%)	0.006
NIHSS score	5.75 ± 5.42	5.93 ± 6.28	6.35 ± 6.61	8.24 ± 6.78	0.009

ALT, alanine aminotransferase; AST, aspartate aminotransferase; BMI, body mass index; SBP, systolic blood pressure; DBP, dilated blood pressure; HbA1c, glycated hemoglobin; LDL-C, low-density lipoprotein cholesterol; HDL-C, high-density lipoprotein cholesterol; TC, total cholesterol; TG, total triglyceride FPG, fasting plasma glucose; GGT, gamma-glutamyltransferase; UA, uric acid; BUN, blood urea nitrogen; Apo B: Apolipoproteins B; ApoA-1: Apolipoproteins A-1; sdLDL-C: Small dense low-density lipoprotein cholesterol; HT, hypertension; Hcys, homocysteine; IAC, intracranial artery calcification; NIHSS, National Institutes of Health Stroke Scale.

**Figure 2:**
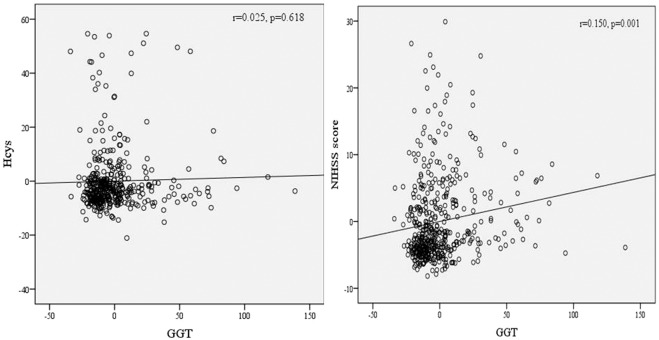
Partial Spearman correlation coefficients showed the association between serum GGT levels with serum homocysteine levels and NIHSS score after adjustment for age and gender.

We analyzed the association between the GGT level to the risk factor of Hcys and the NIHSS score reflecting the prognosis of stroke patients. After adjusting age and gender, partial correlation analysis showed that there was no significant correlation between GGT and Hcys (r = 0.025, p = 0.618, Fig. 2), whereas there was a certain correlation with the NIHSS score (r = 0.150, p = 0.001, Fig. 2).

Logistic regression analysis showed that serum GGT levels predicted short-term all-cause mortality (OR = 1.016, 95% CI: 1.004–1.029, p = 0.012), NIHSS scores (β = 0.035, 95% CI: 0.006–0.064, p = 0.017), and IAC scores (β = 0.009, 95% CI: 0.002–0.015, p = 0.008) in male patients with unadjusted variables (Table 2). After adjustment of age, smoking, drinking, HT, DM, CHD, DBP, TC, TG, Hcys, HbA1c and UA, GGT levels could independently predict the risk of all-cause mortality (OR = 1.036, 95% CI: 1.014–1.060, p = 0.002), NIHSS scores (β = 0.051, 95% CI: 0.020–0.082, p = 0.001) and IAC scores (β = 0.006, 95% CI: 0.003–0.014, p = 0.005) in male patients. However, GGT levels could not independently be associated with all-cause mortality, NIHSS scores and IAC scores in female patients (Table 2).

**Table 2:** Association of serum GGT levels with death, NIHSS score and IAC score according to univariate and multivariate analyses.

Male	Death			NIHSS score			IAC score		
	OR	95% CI	*P* value	β	95% CI	*P* value	β	95% CI	*P* value
unadjusted	1.016	1.004–1.029	0.012	0.035	0.006–0.064	0.017	0.009	0.002–0.015	0.008
Model 1	1.020	1.006–1.034	0.004	0.042	0.012–0.072	0.006	0.008	0.001–0.015	0.017
Model 2	1.036	1.014–1.060	0.002	0.051	0.020–0.082	0.001	0.006	0.003–0.014	0.005
**Female**									
unadjusted	1.010	0.991–1.029	0.300	0.048	−0.006–0.101	0.080	0.008	−0.003–0.019	0.145
Model 1	1.015	0.994–1.036	0.157	0.060	0.007–0.114	0.027	0.007	−0.005–0.018	0.257
Model 2	0.752	0.524–1.078	0.121	0.036	−0.071–0.072	0.991	0.015	−0.003–0.033	0.110

Model 1 adjusted for age, smoking, drinking, HT, DM, CHD; Model 2 adjusted for Model 1 plus DBP, TC, TG, Hcys, HbA1c and UA.

Table 3 shows the relationships between male patient’s serum GGT quartiles and all-cause hospital mortality both in unadjusted and adjusted models. Multivariable logistic regression analysis showed that the risk of all-cause hospital mortality in fourth GGT quartile group was significantly higher than other quartiles, both in univariate analysis (OR = 4.164, 95% CI: 1.157–14.991, *P* = 0.029, *P*_trend_ = 0.031) and after adjustment for the 12 demographic, medical history and laboratory variables (OR = 5.616, 95% CI: 1.339–20.641, *P* = 0.034, *P*_trend_ = 0.034). Each SD increase of serum GGT levels (Z score transformed) was also associated with risk of all-cause hospital mortality, both in univariate analysis (OR = 1.443, 95% CI: 1.085–1.919, *P* = 0.012) and after adjustment (OR = 2.272, 95% CI: 1.364–3.787, *P* = 0.002).

**Table 3:** Odds ratios and 95% confidence intervals of all-cause hospital mortality risk according to serum GGT quartiles in male AIS patients.

Death	Quartile 1	Quartile 2	Quartile 3	Quartile 4	*P*_trend_	Each SD increase of GGT
Unadjusted	1	1.118 (0.257–4.862)	2.500 (0.658–9.501)	4.164 (1.157–14.991)	0.031	1.443 (1.085–1.919)
*P* values		0.882	0.179	0.029		0.012
Model 1	1	1.252 (0.278–5.643)	2.952 (0.745–11.698)	4.785 (1.261–18.156)	0.028	1.589 (1.157–2.182)
*P* values		0.770	0.123	0.021		0.004
Model 2	1	0.895 (0.046–7.432)	3.057 (0.849–14.156)	5.616 (1.339–20.641)	0.034	2.272 (1.364–3.787)
*P* values		0.941	0.153	0.034		0.002

Model 1 adjusted for age, smoking, drinking, HT, DM, CHD; Model 2 adjusted for Model 1 plus DBP, TC, TG, Hcys, HbA1c and UA.

A total of 117 patients were diagnosed with severe IAC. GGT levels in patients with severe IAC were significantly elevated (37.6 ± 33.6 vs 28.6 ± 19.2, p < 0.001). However, NIHSS scores (6.66 ± 5.78 vs 6.53 ± 6.44, p = 0.873) and mortality (8.1% vs 12.4%, p = 0.333) in patients with severe IAC were not significantly different from those in patients with non-severe IAC. Logistic regression analysis showed that after adjusting 12 variables, the GGT level could not predict the severity of IAC (OR = 1.009, 95% CI: 0.994–1.024, p = 0.239). Meanwhile, there was no difference in the risk of male patient’s calcification between different quantiles groups in both unadjusted and adjusted models (Table 4). Each SD increase of serum GGT levels was associated with risk of severe IAC in univariate analysis (OR = 1.409, 95% CI: 1.091–1.820, *P* = 0.009). However, in adjusted models, this association was diminished (OR = 1.230, 95% CI: 0.871–1.735, *P* = 0.239, Table 4).

**Table 4:** Odds ratios and 95% confidence intervals of severe IAC risk according to serum GGT quartiles in male AIS patients.

Several IAC	Quartile 1	Quartile 2	Quartile 3	Quartile 4	*P*_trend_	Each SD increase of GGT
Unadjusted	1	0.548 (0.188–1.601)	1.486 (0.591–3.733)	1.884 (0.770–4.613)	0.054	1.409 (1.091–1.820)
*P* values		0.272	0.400	0.166		0.009
Model 1	1	0.514 (0.174–1.521)	1.365 (0.534–3.489)	1.695 (0.676–4.252)	0.082	1.386 (0.061–1.810)
*P* values		0.229	0.516	0.261		0.017
Model 2	1	0.815 (0.209–3.176)	1.952 (0.604–6.305)	1.874 (0.555–6.321)	0.366	1.230 (0.871–1.735)
*P* values		0.768	0.263	0.311		0.239

Model 1 adjusted for age, smoking, drinking, HT, DM, CHD; Model 2 adjusted for Model 1 plus DBP, TC, TG, Hcys, HbA1c and UA.

## Discussion

This is the first study that assesses the relationship between serum GGT levels and IAC in AIS patients in the Chinese population. In the present study, we found that serum GGT levels were significantly elevated in patients who died. High circulating serum GGT levels in men with AIS are associated with increased all-cause mortality and calcification risk. However, GGT could not independently predict the risk of all-cause mortality and calcification in female patients. In additional, the GGT level could independently predict admission NIHSS scores in male patients with AIS.

Jousilahti *et al*., identified the relationship between serum GGT levels and alcohol consumption in 14,000 stroke patients^10^. However, the author only interprets GGT as an alternative indicator of alcohol consumption. Emdin M. *et al*., offers another explanation for the findings based on previous evidence. Although serum GGT is a good indicator of alcohol abuse, epidemiological studies have shown a positive correlation between GGT level and cardiovascular risk factors, regardless of alcohol consumption^19^. A meta-analysis of 926,497 subjects in 10 prospective studies by Zhang X. W. *et al*. showed that serum GGT level was an independent risk factor for stroke^20^. We found that GGT levels were independently associated with short-term all-cause mortality in men with AIS. Active GGT is present in atherosclerotic plaques^21^. We therefore hypothesized that GGT could be present in cerebral atherosclerotic plaques in patients with AIS. In addition, the GGT levels of patients who died were significantly elevated. Serum GGT level also was independently associated with admission NIHSS score in male patients. We postulated that the GGT enzyme could be associated with the occurrence of an acute stroke event while reflecting the severity of stroke in male.

Several studies have confirmed the relationship between GGT and coronary artery or valve calcification^22^. Cappelli *et al*. demonstrated that the GGT enzyme was concentrated on lipid deposition and stenosis of aortic valve^23^. IAC is useful as an easily accessible biomarker that reflects the severity of intracranial vascular diseases. Eroglu S. *et al*., found that serum GGT activity is connected with carotid intima-media thickness (IMT)^24^. The catalytic GGT enzyme was found to be mainly accumulated in atherosclerotic plaques rich in LDL and CD68^+^ foam cells in cerebral arteries, carotid arteries, and coronary atherosclerotic plaques by endarterectomy^21^. Serum GGT may be involved in the accumulation of GGT in atherosclerotic plaque^21^. GGT can trigger iron-catalyzed LDL oxidation and produce reactive oxygen species, which may promote arterial wall injuries and plaque calcification^25^. In the current study, we report a significant correlation between serum GGT activity and IAC score in male patients. The reference range of GGT activity is similar among different age groups. Although there is significant gender difference, the GGT activity of men is higher than that of the women^26^. We found that each SD increase in male’s serum GGT levels was associated with risk of several IAC in univariate analysis. High GGT activity may be an important risk factor for calcification.

As a widely accepted risk factor for stroke patients, Hcys has been broadly used in clinical practice. We first evaluated the relationship between GGT levels and Hcys in patients with AIS. Previous studies have shown that combinations of GGT and Hcys can be used to assess early nutritional status in patients with alcohol dependence and heart failure^27,28^. Tu W. J. *et al*., demonstrated that low circulating levels of retinoic acid (RA) were associated with increased risk of all-cause and CVD mortality in a cohort of patients with first-incidence AIS^29^. Limpach *et al*., founded that Hcys could inhibit RA synthesis through a specific mechanism for Hcys-induced congenital defects^30^. All-*trans* retinoic acid increased GGT activity in a cell type-and time-dependent manner^31^. Currently, the relationship among GGT, Hcys and RA could not be determined. But an underlying mechanism may exist between them. In the future, assessment of GGT, Hcys and RA in combinations may be useful to determine the early risk of AIS patients, especially in those with alcohol dependence.

As an observational study, this study has the following shortcomings: First, the number of AIS patients involved in this study is less than other studies. In addition, we only retrospectively reviewed the situations of patients’ death at about 60 days; Second, there are currently no suitable quantitative testing, scoring tools and methods for IAC. The risks of IAC and plaque rupture or death in stroke patients remain controversial. To understand if GGT levels could be used as a potential risk factor for CVD patients with cerebrovascular diseases, there is a need for more prospective cohort studies.

## Conclusion

In conclusion, we have evaluated the relationship between serum GGT levels and IAC among AIS patients in the Chinese population. High circulating serum levels of GGT were associated with increased risk of all-cause mortality and IAC in male patients with AIS.
